# Digital twin syncing for autonomous surface vessels using reinforcement learning and nonlinear model predictive control

**DOI:** 10.1038/s41598-025-93635-9

**Published:** 2025-03-18

**Authors:** Henrik Stokland Berg, Daniel Menges, Trym Tengesdal, Adil Rasheed

**Affiliations:** 1 Department of Engineering Cybernetics, Norwegian University of Science and Technology, Trondheim, Norway; 2 Department of Mathematics and Cybernetics, SINTEF Digital, Trondheim, Norway

**Keywords:** Deep reinforcement learning, Nonlinear model predictive control, Autonomous surface vessel, Parameter optimization, Model identification, Computer science, Mechanical engineering

## Abstract

Current control systems for autonomous surface vessels (ASVs) often disregard model uncertainties and the need to adapt dynamically to varying model parameters. This limitation hinders their ability to ensure reliable performance under complex and frequently changing maritime conditions, highlighting the need for more adaptive and robust approaches. Therefore, this study introduces an innovative approach that integrates deep reinforcement learning (DRL) with nonlinear model predictive control (NMPC) to optimize the control performance and model parameters of ASVs. The primary objective is to ensure that the digital twin of the ASV remains continuously synchronized with its physical counterpart, thereby enhancing the accuracy, reliability, and adaptability of the digital twin in representing the vessel under complex and dynamic maritime conditions. Leveraging the capabilities of digital twins, agents can be trained in safety-critical applications within a risk-free virtual environment, minimizing the hazards associated with real-world experimentation. The DRL framework optimizes NMPC by tuning its parameters for peak performance and identifying unknown model parameters in real-time, ensuring precise and dependable vessel control. Extensive simulations confirm the effectiveness of this approach in improving the safety, efficiency, and reliability of ASVs. The proposed methods address critical challenges in ASV control by enhancing reliability and adaptability under dynamic conditions, providing a foundation for future advancements in autonomous maritime navigation and control system development.

## Introduction

In the constantly evolving domain of autonomous systems, the concept of a digital twin has gained substantial traction as an influential technology. The definitions of digital twins differ depending on the discipline and application, but they typically share a focus on combining accurate models with real-time (or near real-time) data streams. These elements are essential for replicating the behavior and dynamics of physical systems. In general, a digital twin can be defined as a virtual representation of a physical asset or a process enabled through data and simulators for real-time monitoring, prediction, optimization, control, and informed decision-making^1^. Based on the capability, a digital twin can be categorized on a scale from 0-5 (0-standalone, 1-descriptive, 2-diagnostic, 3-predictive, 4-prescriptive, 5-autonomous)^2,3^. Central to the effectiveness of digital twins across all capability levels is the process of modeling. Accurate models are critical as they form the foundation upon which digital twins are built. These models encapsulate the behavior, dynamics, and interactions of physical systems, enabling simulations that can predict performance under various conditions. Control strategies, on the other hand, ensure that the system operates within desired parameters by responding to changes in real-time. However, it is crucial to recognize that no model is perfect. The complexity of real-world systems means that models inevitably contain approximations and assumptions. In the context of autonomous surface vessels (ASVs), a drift between the digital twin and its physical counterpart can arise due to environmental and operational factors. For instance, fouling on the hull, such as barnacle growth or algae accumulation, alters hydrodynamic properties, including drag coefficients. This leads to deviations in performance metrics such as fuel efficiency and maneuverability. Similarly, gradual wear on propulsion systems or steering mechanisms can cause discrepancies between the predicted and actual behavior of the vessel. Over time, these discrepancies can cause the digital twin to drift away from accurately representing the physical system. To mitigate this drift, continuous updating and refining of models based on real-time data is essential. This ongoing process ensures that the digital twin remains a faithful and useful representation of the physical asset. However, this approach faces limitations, such as the need for robust computational infrastructure, which may not always be feasible in remote maritime operations. Sensor inaccuracies or failures can worsen drift, adding uncertainty to the digital twin’s predictions. Tackling these issues demands further research into adaptive algorithms and resilient data integration methods. In addition to model updates, it is also necessary to continuously learn and adapt control strategies. As the digital twin evolves, so too must the approaches used to manage and control the system it represents. This learning process can occur in a virtual environment^4,5^, where simulations can explore a wide range of scenarios without risking the physical system. By learning in this way, digital twins can develop robust control strategies that are well-suited to real-world conditions, enhancing their effectiveness and reliability.

Digital twin technology has demonstrated its potential to enhance maritime operations, showcased by TwinPort, which utilizes 5G networks and drone-assisted data collection to improve navigation and efficiency in smart seaports^6^. Similarly, advancements in distributed synchronization methods for underactuated surface vessels have shown promise in addressing challenges such as actuator failures and unknown control directions, enabling robust tracking and connectivity maintenance in dynamic environments^7^. Moreover, recent advancements in cyber-resilient digital twin frameworks, such as the one proposed for 6G internet of vehicles networks, show potential for improving computational efficiency and network security by addressing limitations of static models and high computational demands through advanced feature engineering and online learning^8^. Although cybersecurity remains a critical consideration for digital twin systems, it falls outside the scope of this work.

While digital twins offer significant benefits for autonomous systems, they rely heavily on the accuracy of the underlying models and the effectiveness of control strategies. Without continuous learning and adaptation, both in terms of modeling and control, the digital twin will inevitably diverge from its real-world counterpart, limiting its usefulness and accuracy. To this end, this article evaluates the potential of integrating deep reinforcement learning (DRL) with nonlinear model predictive control (NMPC) to enhance adaptive control mechanisms of autonomous systems. In addition, it explores how such integration can enrich digital twins by continuously updating asset model parameters based on real-time feedback. The asset chosen to model the system is an ASV.

Recently, the capabilities of ASVs have greatly improved through the use of rapidly emerging machine learning and artificial intelligence technologies^9,10^. DRL methods have emerged as a promising alternative for the challenging task of autonomous navigation at sea, making progress in solving long-existing problems of ASVs that have not yet been solved using traditional methods^11^. By learning optimal control policies through trial-and-error interactions with the environment, DRL agents can adapt to complex and dynamic maritime scenarios, showing good performance in these sequential decision-making problems^12^. However, their widespread application to real-world maritime operations remains limited due to the reinforcement learning (RL) algorithms’ “black-box” nature. Concerns regarding the safety verification and explainability of RL-based decision-making processes raise significant challenges for the certification and deployment of these systems in safety-critical maritime environments^10,13^.

While DRL offers adaptability, the interpretability of traditional control methods, where the decision-making process is more transparent and explainable, can simplify the certification process for safety-critical maritime applications^14^. This inherent explainability makes verifying the safety and reliability of such controllers easier. Several studies have demonstrated that model predictive control (MPC) and its nonlinear variant, NMPC, offer effective solutions for collision avoidance (COLAV) of ASVs. In^15^, a scenario-based MPC for COLAV is proposed incorporating COLREG and extended in^16^ to a probabilistic and intention-aware version able to consider uncertain obstacle kinematics. In addition, an NMPC method for trajectory tracking and COLAV is developed by^17^, utilizing elliptical ship domains and a nonlinear disturbance observer for enhanced robustness but depend on other vessels’ velocity, course, and length data, which are often unavailable.

Furthermore, MPC, combined with potential fields, has proven effective in other autonomous systems, such as autonomous vehicles. For example, in^18^, an adaptive potential field for connected autonomous vehicles is introduced, improving computation time by decoupling the APF from the MPC, while in^19^, an optimal path planning technique for autonomous vehicles in dynamic environments is demonstrated, integrating MPC for lane-keeping and lane-changing decisions using distinct potential field functions for road boundaries and obstacles. Another proposal combines a potential field-based extended dynamic window approach with linear MPC for automated berthing, suitable for mild environmental conditions^20^.

Given the lack of robust control schemes addressing COLAV, anti-grounding, and trajectory tracking under environmental disturbances, the NMPC method proposed in^21^ shows suitability in tackling COLAV and path following by utilizing artificial potential fields (APFs) and an environmental disturbance observer^22^. Although the study demonstrates promising results, optimization and fine-tuning of this approach still remain a challenge. In this context, one of the major challenges of NMPC concerning autonomous applications is the non-trivial tuning process of parameters influencing the control performance while crafting the objective function of the optimal control problem (OCP). The weights assigned to different objectives can significantly impact the performance of the controller^23^. Tuning parameterization is related to the process behavior, making their determination a complex task^24^. The tuning process is often executed using a trial-and-error method, which is a cumbersome task due to the overlapping effects of the tuning parameters and nonlinearity brought by the constraints of the OCP^25^. For NMPCs, the complexity increases due to the need to handle nonlinear dynamics and constraints, which are more difficult to model and predict accurately compared to linear systems. Additionally, the interactions between different parameters are more pronounced in NMPC, increasing the complexity of manual tuning^26^. Several tuning guidelines for MPC can be found in the literature^23,27^. In^23^, an overview of the tuning of linear MPC controllers is given, and in^28^, a procedure for tuning NMPC on multi-objective optimization methods is presented.

Genetic algorithms (GAs) have demonstrated effectiveness in real-time weight optimization for NMPC in ship trajectory tracking^29^, and particle swarm optimization (PSO) has proven effective for receding horizon control in complex systems such as district heating networks^30^. In comparison, DRL inherently captures temporal dependencies through its reward-based learning framework and optimizes long-term performance, which is critical for sequential decision-making tasks in dynamic environments. DRL has shown promising results in tuning MPC controllers. In^31^, the optimization of MPC meta-parameters is explored using reinforcement learning to enhance both control performance and computational efficiency. The study proposes a novel framework where RL optimizes any control algorithm parameters, demonstrating significant improvements in the control of an inverted pendulum system, achieving a 36% reduction in computation time and an 18.4% improvement in control performance. Another study shown in^32^ highlights the potential of using DRL to optimize the parameters of NMPCs, particularly emphasizing the importance of meta-parameter optimization in MPC schemes and how RL can be utilized to achieve this. A high-level trajectory planning algorithm for autonomous quadrotors based on MPC tuned with machine learning is presented in^33^. This method successfully guided a quadcopter to its target while avoiding obstacles, demonstrating the effectiveness of using machine learning to tune MPC parameters for unmanned aerial vehicle operations involving obstacles. Additionally, in^34^, an MPC-based RL method is used to optimize the control policy for ASVs, achieving improved closed-loop performance in collision-free path following and autonomous docking.

However, MPC is a model-based control scheme that requires an accurate model of the system to achieve satisfactory control behavior^35^. The need for accurate modeling of ship dynamics is also highlighted by the International Maritime Organization (IMO)^36^, which endorses using mathematical models and simulations for predicting ship performance at the design stage. Several methods can be employed to determine the model parameters and coefficients in mathematical ship maneuvering models^37,38^. These include using empirical formulas, captive model tests, computational fluid dynamics (CFD)^39,40^, and system identification (SI) combined with free-running model tests or full-scale experiments. System identification has emerged as a valuable tool for establishing mathematical models of ship motion due to its efficiency and relative simplicity^41–44^.

The limitations of these traditional and advanced techniques create a knowledge gap in the field of ASV control. This study aims to address this gap by exploring the potential of DRL in conjunction with NMPC for online model parameter identification. By using the adaptive learning capabilities of DRL, the goal is to develop a framework that can estimate model parameters in real-time, potentially overcoming the limitations of existing methods and leading to more accurate and efficient control strategies for ASVs. This approach is particularly well-suited for scenarios where the ASV model is initially uncertain or subject to change due to varying operating conditions or environmental factors. By continuously adapting the model parameters based on real-time feedback, the DRL-NMPC framework can maintain accurate predictions and ensure optimal control performance.

Summarized, this work explores the integration of DRL with NMPC to develop a robust control framework for ASVs. By leveraging DRL for NMPC performance optimization and model parameter identification, the proposed approach aims to enhance the navigational performance, safety, and reliability of ASVs in complex and dynamic maritime environments.

## Theory

This section presents the vessel dynamics employed in this study and the formulation of the NMPC problem, which serves as the foundation for DRL-based parameter optimization.

### Vessel model dynamics

In this section, the model dynamics used for the ASV are outlined. The model is adopted from^37^ and offers a thorough derivation of modeling and controlling of marine vessel dynamics. The ship model is a 3 degrees of freedom (DOF) model. The 3 kinematic states of the vessel are $\varvec{\eta } = [x_{s}, y_{s}, \psi ]^{\top }$, where $x_{s}$ and $y_{s}$ are the position of the vessel relative to the global coordinates $[x, y]^{\top }$ and $\psi$ is the heading angle of the vessel. The global coordinates are defined in the north-east-down (NED) coordinate system. Moreover, the velocities of the vessel in the BODY-fixed coordinate system are given by $\varvec{\nu } = [u, v, r]^{\top }$, where *u* is the surge velocity, *v* is the sway velocity, and *r* is the yaw rate. Using these relationships, the kinematics of a vessel can be expressed by

1 $$\begin{aligned} \dot{\varvec{\eta }} = {\textbf{R}}_{\textrm{rot}}(\psi ) \varvec{\nu }, \end{aligned}$$

where ${\textbf{R}}_{\textrm{rot}}(\psi )$ denotes the rotational matrix from the NED coordinate system $\{n\}$ to the BODY frame $\{b\}$, given by

2 $$\begin{aligned} {\textbf{R}}_{\textrm{rot}} = {\textbf{R}}^{n}_{b} = \begin{bmatrix} \cos (\psi ) & -\sin (\psi ) & 0 \\ \sin (\psi ) & \cos (\psi ) & 0 \\ 0 & 0 & 1 \end{bmatrix}. \end{aligned}$$

**Figure 1:**
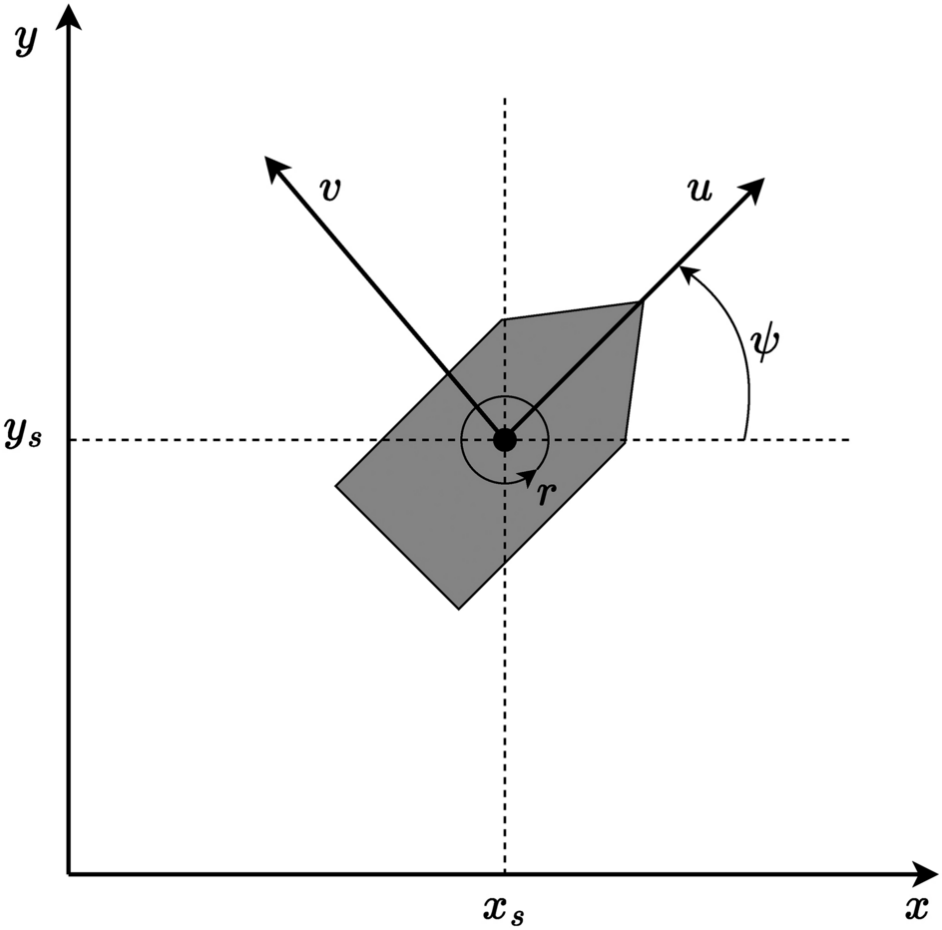
Kinematics of a vessel^22^.

Figure 1 illustrates the relations between the different vessel states. The dynamics of a surface vessel can be formulated as

3 $$\begin{aligned} {\textbf{M}} \dot{\varvec{\nu }} + {\textbf{D}}(\varvec{\nu }) \varvec{\nu } + {\textbf{C}}(\varvec{\nu }) \varvec{\nu } = \varvec{\tau } + \varvec{\tau _{d}}, \end{aligned}$$

where ${\textbf{M}}$ expresses the mass matrix, ${\textbf{D}}(\varvec{\nu })$ describes the nonlinear damping matrix, ${\textbf{C}}(\varvec{\nu })$ denotes the Coriolis and centripetal matrix, $\varvec{\tau }$ is the control input and $\varvec{\tau _{d}}$ describes all the environmental disturbances, driven by wind, waves, and sea currents.

Expressing the vessel dynamics in state space representation results in

4 $$\begin{aligned} \dot{\varvec{\nu }} = {\textbf{M}}^{-1}(-{\textbf{D}}(\varvec{\nu }) \varvec{\nu } - {\textbf{C}}(\varvec{\nu }) \varvec{\nu } + \varvec{\tau } + \varvec{\tau _{d}}). \end{aligned}$$

Further rewriting in control-theoretical notation gives the following state space model

5 $$\begin{aligned} \dot{{\textbf{x}}} = \begin{bmatrix} {\varvec{0}}_{3x3} & {\textbf{R}}_{\textrm{rot}}({\textbf{x}}) \\ {\varvec{0}}_{3x3} & {\textbf{M}}^{-1}[-{\textbf{D}}({\textbf{x}}) - {\textbf{C}}({\textbf{x}})] \\ \end{bmatrix} {\textbf{x}} + \begin{bmatrix} {\varvec{0}}_{3x3} \\ {\textbf{M}}^{-1} \\ \end{bmatrix} {\textbf{u}} + \begin{bmatrix} {\varvec{0}}_{3x3} \\ {\textbf{M}}^{-1} \\ \end{bmatrix} {\textbf{d}}, \end{aligned}$$

where

6 $$\begin{aligned} {\textbf{x}}&= [\varvec{\eta }, \varvec{\nu }]^{\top } = [x_{s}, y_{s}, \psi , u, v, r]^{\top }, \end{aligned}$$

7 $$\begin{aligned} {\textbf{u}}&= \varvec{\tau } = [\tau _{u}, 0, \tau _{r}]^{\top }, \end{aligned}$$

8 $$\begin{aligned} {\textbf{d}}&= \varvec{\tau _{d}} = [\tau _{d,1}, \tau _{d,2}, \tau _{d,3}]^{\top }. \end{aligned}$$

This yields under-actuated vessel dynamics with control inputs defined as thrust in surge ($\tau _{u}$) and moment regarding yaw ($\tau _{r}$), while the components of the environmental disturbances $\tau _{d,1}$, $\tau _{d,2}$, and $\tau _{d,3}$ are forces and moments with regard to the 3 DOF local body frame of the vessel.

### Nonlinear model predictive control (NMPC)

The NMPC used for this study is adopted from^21^. To solve the Optimal Control Problem (OCP) of the NMPC, direct multiple shooting^45^ is used. Defining the system dynamics $\dot{{\textbf{x}}} = f({\textbf{x}}, {\textbf{u}}, {\textbf{d}})$ as in equation (5), and representing the state error as

9 $$\begin{aligned} \tilde{{\textbf{x}}} = \begin{bmatrix} 0 \\ 0 \\ \psi _{\text {des}} - \psi \\ u_{\text {des}} - u \\ 0 \\ r \\ \end{bmatrix}, \end{aligned}$$

where $\tilde{{\textbf{x}}}$ encapsulates the deviations from the desired heading $\psi _{\text {des}}$, desired surge speed $u_{\text {des}}$, and yaw rate. The OCP is then formulated by

10 $$\begin{aligned} \begin{aligned} \min _{{\textbf{u}}(t), {\textbf{x}}(t)}&J({\textbf{x}}(t), {\textbf{u}}(t)) = \sum _{k=0}^{N-1} \tilde{{\textbf{x}}}_k^\top {\textbf{Q}} \tilde{{\textbf{x}}}_k + {\textbf{u}}_k^\top {\textbf{R}} {\textbf{u}}_k + \varvec{\xi }_k^\top {\textbf{W}} \varvec{\xi }_k \\ \text { s.t. } (a)&\quad {\textbf{x}}_{k+1} = f({\textbf{x}}_k, {\textbf{u}}_k, {\textbf{d}}_k), \quad \forall k \in [0, N-1] \\ (b)&\quad {\textbf{x}}_0 = {\textbf{x}}(t) \\ (c)&\quad {\textbf{x}}_{\text {lb}} \le {\textbf{x}}_k \le {\textbf{x}}_{\text {ub}}, \quad \forall k \in [0, N] \\ (d)&\quad {\textbf{u}}_{\text {lb}} \le {\textbf{u}}_k \le {\textbf{u}}_{\text {ub}}, \quad \forall k \in [0, N-1] \\ (e)&\quad {\textbf{d}}_k = \hat{\varvec{\tau }}_d, \quad \forall k \in [0, N-1] \\ (f)&\quad -\varvec{\xi }_k \le \tilde{{\textbf{x}}}_k \le \varvec{\xi }_k, \quad \forall k \in [0, N-1] \\ (g)&\quad \varvec{\xi }_k \ge 0, \quad \forall k \in [0, N-1] \end{aligned} \end{aligned}$$

Here, ${\textbf{Q}}$ is the matrix weighting state errors, ${\textbf{R}}$ weights the control inputs, and $\varvec{\xi }$ comprises slack variables, weighted by ${\textbf{W}}$. The bounds ${\textbf{x}}_{\text {lb}}, {\textbf{x}}_{\text {ub}}, {\textbf{u}}_{\text {lb}},$ and ${\textbf{u}}_{\text {ub}}$ are the lower and upper bounds for states and control inputs and *N* is the prediction horizon. Constraint (*e*) in Eq. (10) indicates that the model dynamics in (*a*) are supplemented by estimated disturbances from a disturbance observer detailed in^22^, facilitating adaptive correction of control inputs in response to external forces. After solving the optimization problem at each time step, the first control input in the optimized input sequence is applied to the system. This approach allows for the predicted states and optimal inputs to be adjusted at the next time step when the open loop optimization problem is solved again with updated state information. This continuous re-optimization with feedback closes the loop and enables the MPC trajectory tracking controller to adapt to any disturbances or deviations in the system dynamics^46^.

## Methodology

This section presents the novel contributions and methods of this study. Therefore, DRL-driven approaches are employed to optimize NMPC parameters and to approximate model parameters for system identification.

### Deep reinforcement learning (DRL) framework

Both the NMPC parameters and the model parameters are determined using identical observation spaces and reward function formulations. Consequently, the subsequent sections detail their respective developments.

#### Observation space

The observation space ${\mathcal {O}}$ for the DRL agent consists of the vessel’s states ${\textbf{x}}$ as shown in Eq. (6), the cross-track error (CTE) $\epsilon (t)$, the desired heading $\psi _{\text {des}}$, the desired surge speed $u_{\text {des}}$, the heading error ${\tilde{\psi }}$, and the surge speed error ${\tilde{u}}$ given by the NMPC. This observation space ensures that the DRL agent has the necessary information about the ASV’s current and desired state, as well as the discrepancies between them, giving an observation of the control performance of the NMPC. Therefore, the observation space is formulated by

11 $$\begin{aligned} \begin{aligned} {\mathcal {O}}&= [{\textbf{x}}, \epsilon (t), \psi _{\text {des}}, u_{\text {des}}, {\tilde{\psi }}, {\tilde{u}} ]\\&=[x_{s}, y_{s}, \psi , u, v, r, \epsilon (t),\psi _{\text {des}}, u_{\text {des}}, {\tilde{\psi }}, {\tilde{u}}]. \end{aligned} \end{aligned}$$

The observation space is normalized to be within the range $[-1,1]$. This is done to ensure efficient and stable learning during training. In real-world applications, these observations are practical and easily realizable. These metrics are typically measured using onboard sensors and navigational systems, ensuring that the transition from a purely NMPC-based system to one enhanced by DRL does not introduce significant additional complexity or cost.

#### Reward function

The reward assignment is crucial for guiding a DRL agent to learn a good policy, as DRL algorithms aim to maximize the reward function, which encapsulates criteria for good and bad actions. A well-formulated reward function is essential for desired behavior learning^47^.

This study uses dense reward functions due to the long time horizons and numerous potential failures per episode. The primary objective is to enhance NMPC path-following capabilities, and dense rewards provide continuous feedback, enabling incremental adjustments for accurate trajectory following. Dense rewards are necessary to handle solver errors in NMPC and help the agent adapt and mitigate these effectively.

The overall reward function *r*(*t*) consists of 4 components aiming for: rewarding for proper path following; punishing for solver errors with respect to the OCP of the NMPC; rewarding if a waypoint is reached; and punishing if a waypoint is passed. Hence, the reward function is defined by

12 $$\begin{aligned}&r_{\text {path}}(t) = \underbrace{\frac{u(t)}{u_{\text {des}}(t)}}_{\text {Speed term}} \cdot \underbrace{\left( \frac{1 + \cos {\tilde{\psi }}(t)}{2}\right) }_{\text {Heading term}} \cdot \underbrace{\left( \frac{1}{\left| \epsilon (t) \right| + 1} \right) }_{\text {CTE term}} \end{aligned}$$

13 $$\begin{aligned}&r_{\text {solver\_error}}(t) = {\left\{ \begin{array}{ll} \frac{\lambda _1}{\left( \frac{t}{t_{\max }} \right) ^2 + \gamma _1} & \text {if solver error occurs} \\ 0 & \text {otherwise} \end{array}\right. } \end{aligned}$$

14 $$\begin{aligned}&r_{\text {goal\_reached}}(t) = {\left\{ \begin{array}{ll} \frac{\lambda _2}{\epsilon (t)^2 + \gamma _2} & \text {if goal is reached} \\ 0 & \text {otherwise} \end{array}\right. } \end{aligned}$$

15 $$\begin{aligned}&r_{\text {goal\_passed}}(t) = {\left\{ \begin{array}{ll} \lambda _3 \cdot \epsilon (t)^2 & \text {if goal passed} \\ 0 & \text {otherwise} \end{array}\right. }\end{aligned}$$

16 $$\begin{aligned}&r(t) = r_{\text {path}}(t) + r_{\text {solver\_error}} + r_{\text {goal\_reached}} + r_{\text {goal\_passed}} \end{aligned}$$

Here, $\gamma _1$ and $\gamma _2$ are small positive numbers added to avoid division by zeros, $\epsilon (t)$ denotes the cross track error at time *t*, and $t_{\text {max}}$ is the maximum time that can be used in each episode. The constants $\lambda _1<<0$, $\lambda _2>0$, and $\lambda _3<0$ are scaling parameters, where solver errors are strongly punished via $\lambda _1$.

### DRL-tuning of NMPC parameters

The main objective of this section is to optimize the design matrices ${\textbf{Q}}$, ${\textbf{R}}$, and ${\textbf{W}}$ of the NMPC. Therefore, a well-defined formulation of the action space is essential.

#### Action space

The action space ${\mathcal {A}}$ comprises the parameterization of the weight matrices ${\textbf{Q}}$, ${\textbf{R}}$, and ${\textbf{W}}$ within the cost function of (10). The action space, similar to the observation space, is normalized to the range [0, 1] to ensure consistency and stability during the learning process. The weight matrices ${\textbf{Q}}$, ${\textbf{R}}$ and ${\textbf{W}}$ are then adjusted in the cost function of the OCP according to

17 $$\begin{aligned}&{\textbf{Q}}_{\text {new}} = {\textbf{Q}}_{\text {lb}} + {\textbf{a}}_{{\textbf{Q}}} \mathbin {\odot }({\textbf{Q}}_{\text {ub}} - {\textbf{Q}}_{\text {lb}}), \end{aligned}$$

18 $$\begin{aligned}&{\textbf{R}}_{\text {new}} = {\textbf{R}}_{\text {lb}} + {\textbf{a}}_{{\textbf{R}}} \mathbin {\odot }({\textbf{R}}_{\text {ub}} - {\textbf{R}}_{\text {lb}}), \end{aligned}$$

19 $$\begin{aligned}&{\textbf{W}}_{\text {new}} = {\textbf{W}}_{\text {lb}} + {\textbf{a}}_{{\textbf{W}}} \mathbin {\odot }({\textbf{W}}_{\text {ub}} - {\textbf{W}}_{\text {lb}}). \end{aligned}$$

Here, ${\textbf{a}}_{{\textbf{Q}}}$, ${\textbf{a}}_{{\textbf{R}}}$ and ${\textbf{a}}_{{\textbf{W}}}$ are the normalized actions from the DRL agent for ${\textbf{Q}}$, ${\textbf{R}}$, and ${\textbf{W}}$, respectively. Note that $\mathbin {\odot }$ represents the Hadamard product (element-wise product). Each weight matrix’s lower and upper bounds, denoted as $(\cdot )_{\text {lb}}$ and $(\cdot )_{\text {ub}}$, are based on the initial guess of the MPC parameters, which were predefined by manual tuning within the digital twin environment. This action space allows safer and faster DRL tuning since it reduces weight exploration concerning unsafe control actions.

### DRL-tuning of model parameters

#### Model parameters

For this study, the Telemetron vessel model is used. Telemetron was an autonomous research vessel operated and owned by Maritime Robotics. The damping matrix consists of linear, quadratic, and cubic components given by

20 $$\begin{aligned} {\textbf{D}}(\varvec{\nu }) = {\textbf{D}}_l + {\textbf{D}}_q(\left| \varvec{\nu } \right| ) + {\textbf{D}}_c(\varvec{\nu }^{\top }\varvec{\nu }), \end{aligned}$$

where

21 $$\begin{aligned}&{\textbf{D}}_l = \begin{bmatrix} D_{l,u} & 0 & 0 \\ 0 & D_{l,v} & 0 \\ 0 & 0 & D_{l,r} \end{bmatrix}, \end{aligned}$$

22 $$\begin{aligned}&{\textbf{D}}_q(\left| \varvec{\nu } \right| ) = \begin{bmatrix} D_{q,u} \left| u \right| & 0 & 0 \\ 0 & D_{q,v} \left| v \right| & 0 \\ 0 & 0 & D_{q,r} \left| r \right| \end{bmatrix}, \end{aligned}$$

23 $$\begin{aligned}&{\textbf{D}}_c(\varvec{\nu }^{\top }\varvec{\nu }) = \begin{bmatrix} D_{c,u} u^2 & 0 & 0 \\ 0 & D_{c,v} v^2 & 0 \\ 0 & 0 & D_{c,r} r^2 \end{bmatrix}. \end{aligned}$$

Here ${\textbf{D}}_l$ is the linear damping term, ${\textbf{D}}_q(\left| \varvec{\nu } \right| )$ is the quadratic damping term, and ${\textbf{D}}_c(\varvec{\nu }^{\top }\varvec{\nu })$ is the cubic damping term, resulting in nonlinear ship dynamics. The Coriolis and centrifugal matrix ${\textbf{C}}(\varvec{\nu })$ of the Telemetron vessel is computed from the mass matrix, following^37^, and is defined by

24 $$\begin{aligned} {\textbf{C}}(\varvec{\nu }) = \begin{bmatrix} 0 & 0 & -m_{22} v - m_{23} r \\ 0 & 0 & m_{11} u \\ m_{22} v + m_{23} r & -m_{11} u & 0 \end{bmatrix}, \end{aligned}$$

where $m_{11}$, $m_{22}$ and $m_{23}$ are elements of the mass matrix ${\textbf{M}}$, defined by

25 $$\begin{aligned} \textrm{M} = \begin{bmatrix} m_{11} & & 0 & & 0\\ 0 & & m_{22} & & m_{23}\\ 0 & & m_{32} & & m_{33} \end{bmatrix}. \end{aligned}$$

Table 5 lists the specific parameter values used in the simulations.

#### Action space

The action space ${\mathcal {A}}$ of the DRL agent, which is used to learn the model parameters of the Telemetron vessel, involves setting the values for the mass matrix ${\textbf{M}}$ and the nonlinear damping matrix ${\textbf{D}}(\varvec{\nu })$ in the NMPC solver. The Coriolis matrix ${\textbf{C}}(\varvec{\nu })$ is calculated from the elements of ${\textbf{M}}$, as detailed in Eq. (24). To facilitate effective operation of the DRL algorithm, the action space is normalized within the interval $[-1, 1]$. Once the normalized action is determined by the DRL agent, it is rescaled within the simulation environment to fit within a specific action range coefficient $\alpha$ of the original model parameters. The mass matrix ${\textbf{M}}$ and the nonlinear damping matrix ${\textbf{D}}(\varvec{\nu })$ are then adjusted in the environment according to

26 $$\begin{aligned}&{\textbf{M}}_{\text {new}} = {\textbf{M}}_{\text {original}} + \alpha \left( {\textbf{a}}_{{\textbf{M}}} \mathbin {\odot }{\textbf{M}}_{\text {original}}\right) ,\end{aligned}$$

27 $$\begin{aligned}&{\textbf{D}}_{\text {new}}(\varvec{\nu }) = {\textbf{D}}_{\text {original}}(\varvec{\nu }) + \alpha \left( {\textbf{a}}_{{\textbf{D}}} \mathbin {\odot }{\textbf{D}}_{\text {original}}(\varvec{\nu })\right) . \end{aligned}$$

Here, ${\textbf{a}}_{{\textbf{M}}}$ and ${\textbf{a}}_{{\textbf{D}}}$ are the normalized actions of the DRL agent for the mass matrix and damping matrix, respectively. Given that certain model parameters of ${\textbf{D}}_c$ and ${\textbf{D}}_q$ were initially identified as 0, they were scaled by the factor 1000 within the action space for higher model sensitivity.

**Figure 2:**
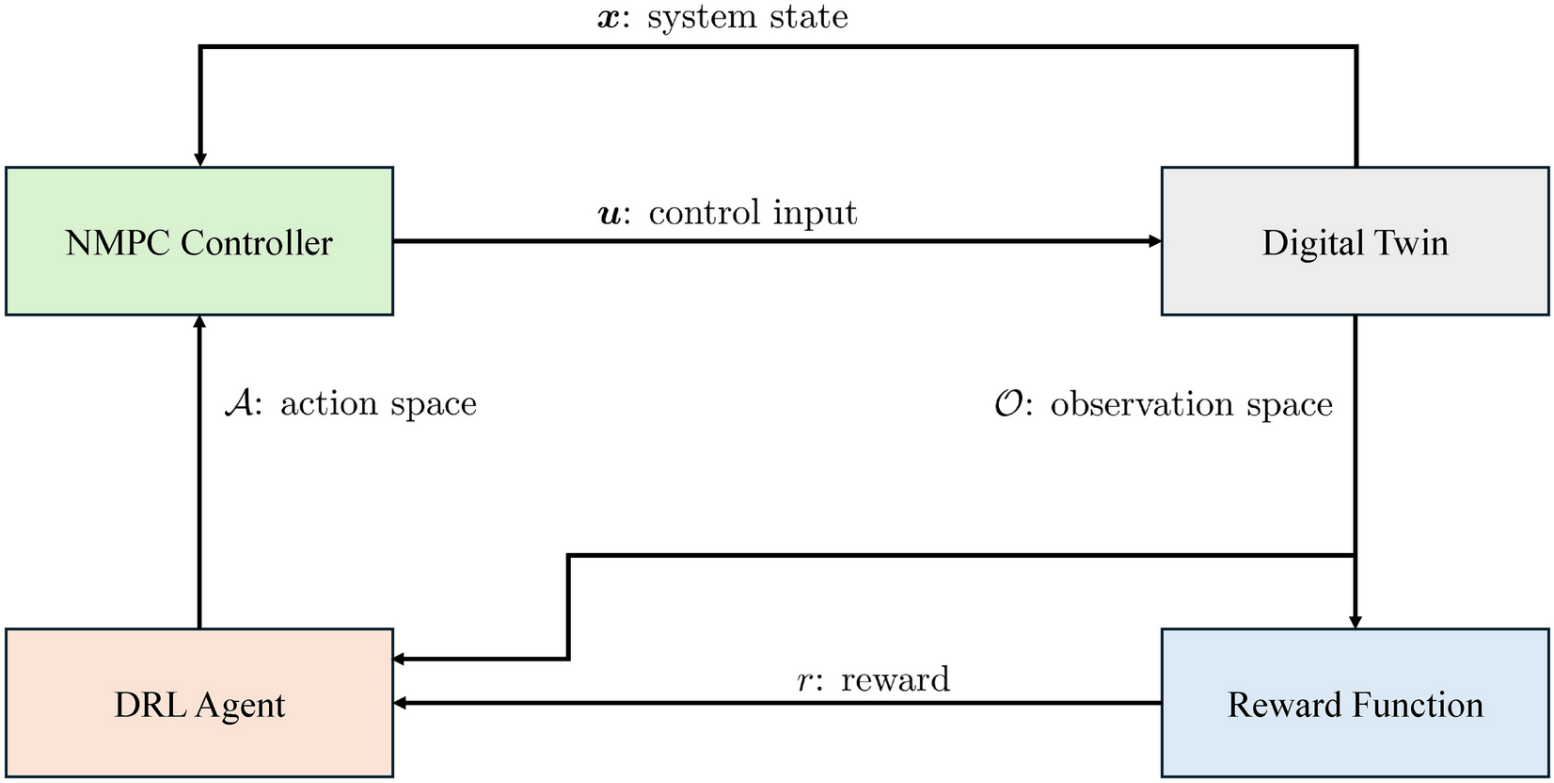
Block diagram of the methodology for optimizing NMPC and model parameters using DRL.

Initially, setting the action range coefficient $\alpha =1$ resulted in an action space spanning from 0 to twice the value of the actual model parameters. This approach proved unsuccessful in training the DRL agent, as it failed to discover reasonable values for the model parameters. Consequently, persistent solver errors occurred in the numerical solver of the NMPC. Subsequently, the action range coefficient $\alpha$ was systematically reduced until successful convergence of the DRL training was observed with $\alpha = 0.25$, corresponding to an action space spanning ± 25 $\%$ of the original values. Subsequent efforts focused on accurately estimating the model parameters within a predefined range of values. The entire process is visually illustrated in Fig. 2, showing that the NMPC and model parameters, which form the action space together, are updated at each time step.

## Results and discussion

In this section, the results of the study on the performance of DRL-tuned NMPC ASVs for path-following and collision avoidance (COLAV) tasks are discussed. The simulations utilized the framework developed by^48^, enabling realistic scenarios that integrate real-world sea mapping through electronic navigational charts (ENCs) and function as a digital twin, incorporating vessel models and physics-based representations of environmental factors such as wind and waves. The analysis covers various scenarios, including operations without environmental disturbances and those with disturbances. Moreover, the effectiveness of DRL tuning on different performance metrics is evaluated, comparing it with baseline NMPC parameters. The results provide insights into the robustness and efficiency of the DRL-tuned NMPC in dynamic and challenging maritime environments. Subsequently, it is demonstrated how DRL can be utilized to approximate model parameters of uncertain model dynamics.

The training was conducted on hardware equipped with an NVIDIA GeForce RTX 4090 GPU and a 13th Gen Intel(R) 32-Core i9-13900KF processor. The prediction horizon of the NMPC was set to $N=10$ for the DRL training and evaluation, with a time step $\Delta t=1 s$ in both the simulator and the NMPC controller. This setting did not cause significant performance degradation but resulted in substantial reductions in training time by decreasing the computational load of the CasADi optimization solver and overall simulation time in the digital twin framework. The training time for simulating 5 million time steps, each with a step size of 1 second, was approximately one day. This corresponds to an average computation time of 0.01728 seconds per time step. The process could be drastically accelerated by parallelizing it through GPUs, enabling increased complexity and extended prediction horizons. Considering multiple agents in the proposed framework, each ASV’s NMPC and model updates scale independently of the fleet size in a decentralized setup, enabling near-linear scalability with sufficient computational resources. However, increased environmental complexity or interconnected agents can affect NMPC convergence, potentially increasing computational demand.

### DRL-tuned NMPC parameters

#### Analysis without disturbances

**Figure 3:**
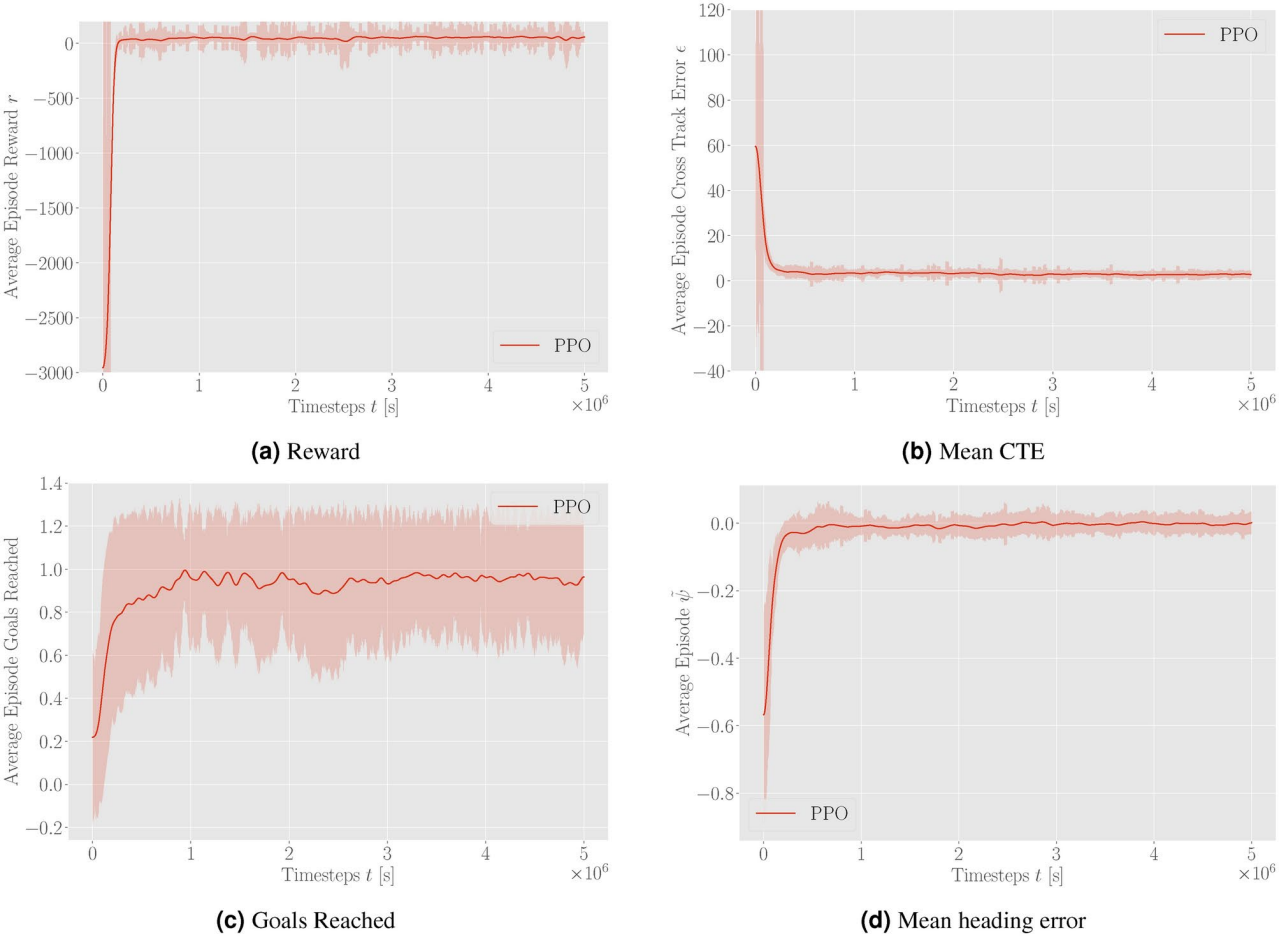
Average episode reward, CTE, goals reached, and heading error during training without the inclusion of environmental disturbances smoothed with a rolling average over 100 episodes.

Figure 3 presents the performance increase of the DRL agent during training using proximal policy optimization (PPO).

**Figure 4:**
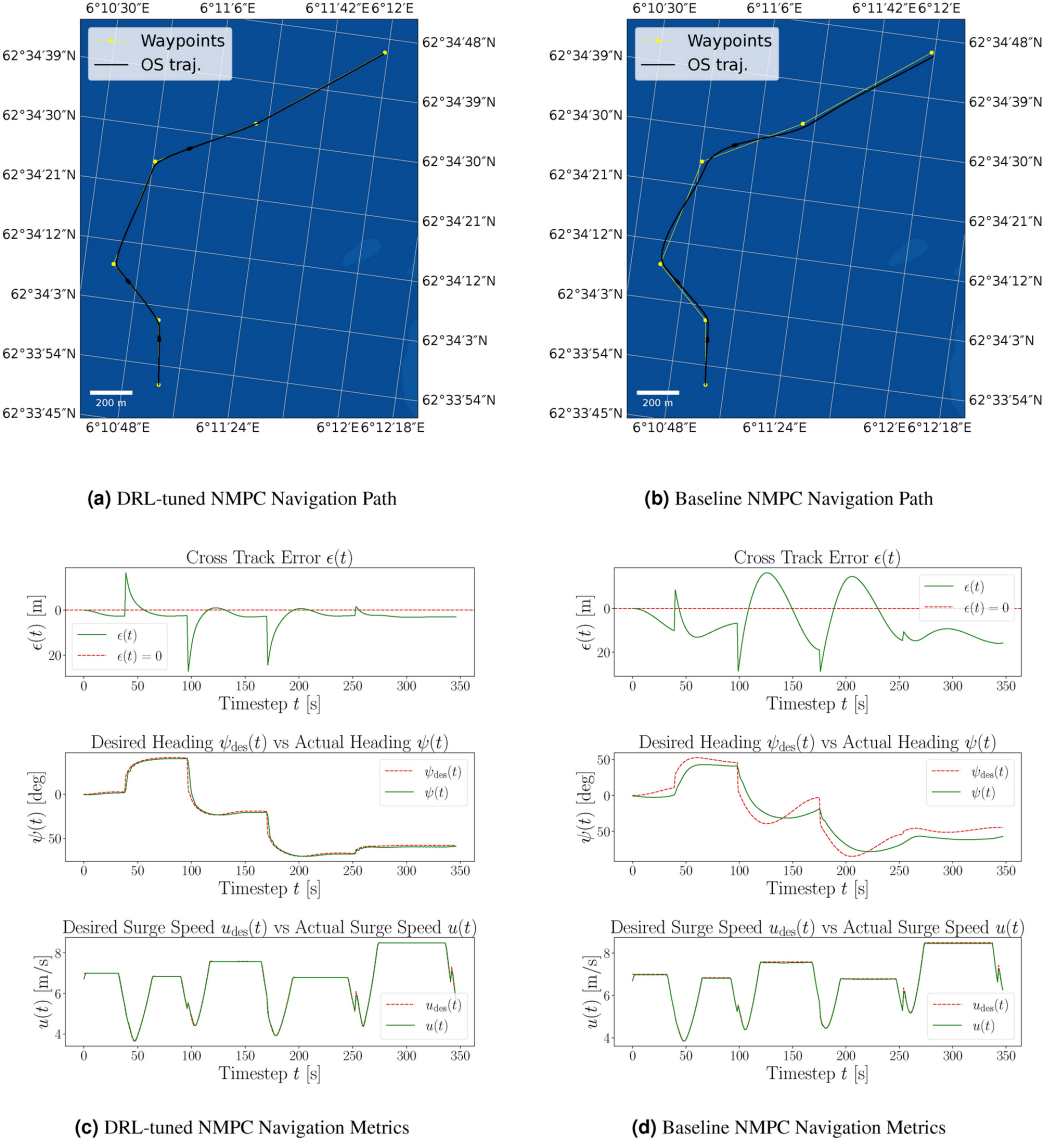
Comparison of path following in the same example scenario for DRL-tuned NMPC and baseline NMPC. The yellow dotted line represents the desired path, and the black line shows the actual path of the ASV.

The approach using DRL significantly improves the performance of the ASV agent by reducing the CTE and heading error, leading to more precise navigation. Additionally, the DRL-tuned agent achieves a higher percentage of goals reached, demonstrating enhanced overall effectiveness in path-following tasks. The agent displays convergence towards the near-maximum value of the reward function. During the initial stages of the DRL training, the average reward is substantially negative, indicating that the DRL agent is tuning the NMPC in a manner that results in solver errors, as the agent is heavily penalized in this case. This initial negative trend is expected as the agent explores various strategies to minimize the overall cost function. However, the convergence to positive values with comparatively low standard deviation suggests that the DRL agent successfully learns to tune the NMPC to find feasible solutions of the OCP that simultaneously maximizes the cumulative reward. The positive rewards indicate that the agent is effectively improving the control performance, stabilizing around a high reward value, which signifies successful policy learning. A pre-tuned (baseline) NMPC was used to compare it to the DRL-tuned agent. For that purpose, several randomly generated scenarios were evaluated to assess and contrast the performance metrics of both approaches, depicted in Table 1. The performance comparison of the DRL-tuned NMPC for a standard path-following scenario is visualized in Fig. 4. Here, Fig. 4a demonstrates the navigation path of the DRL-tuned NMPC, closely following the desired path with minimal cross-track error. Figure 4b shows the navigation path of the NMPC with initial guess parameters, displaying significant deviations and oscillations. Figure 4c details the navigation metrics for the DRL-tuned NMPC, including trajectory, CTE, desired vs. actual heading, and desired surge speed vs. actual surge speed over time. Figure 4d presents the same metrics for the NMPC with initial guess parameters, highlighting the larger CTE, heading error, and inconsistencies in maintaining the desired path.

The DRL-tuned NMPC achieves a significantly higher average reward of 97.53 compared to the baseline NMPC’s -669.96, with a notably lower standard deviation, highlighting its enhanced effectiveness in maximizing rewards and path-following performance. Furthermore, the DRL approach substantially reduces the average CTE to 2.38 meters from the baseline’s 10.36 meters, indicating closer tracking to the desired path with more consistent performance and lower deviations. In terms of goal-reaching, the DRL-tuned NMPC achieves a 100% success rate compared to the baseline’s 27%, demonstrating its superior reliability and robustness. The DRL-tuned controller also shows markedly reduced heading errors and more stable navigation, with lower standard deviation in heading error metrics. While surge speed errors are close to zero for both controllers, the DRL-tuned NMPC shows slightly lower standard deviation and maximum errors, indicating improved speed control. Overall, the evaluation metrics demonstrate that the DRL-tuned NMPC outperforms the baseline NMPC across various performance metrics, enhancing accuracy, reliability, and efficiency in path-following tasks. Despite the baseline NMPC being optimized for simpler scenarios, the DRL-tuned NMPC’s superior performance underscores the potential benefits of using DRL for tuning NMPCs in more challenging scenarios.

**Table 1:** Comparison of DRL-tuned NMPC and baseline NMPC performance evaluated over 100 random generated evaluation episodes with the same scenario configuration as in training, but different seed for random generation.

Metric	DRL-tuned NMPC	Baseline NMPC
Episodes	100.00	100.00
Avg. reward	97.53	-669.96
Std. reward	107.10	719.89
Avg. cross-track error [m]	2.38	10.36
Avg. std cross-track error [m]	4.29	12.67
Avg. max cross-track error [m]	23.99	38.95
Avg. time steps [s]	350.27	358.10
Goals reached (%)	100.00%	27.00%
Infeasible solutions	0.0	0.0
Avg. heading error [deg]	0.02	0.13
Avg. std heading error [deg]	0.08	0.18
Avg. max heading error [deg]	0.28	0.42
Avg. surge speed error [m/s]	0.00	0.07
Avg. std surge speed error [m/s]	0.21	0.19
Avg. max surge speed error [m/s]	1.43	1.49

#### Analysis including disturbances

**Figure 5:**
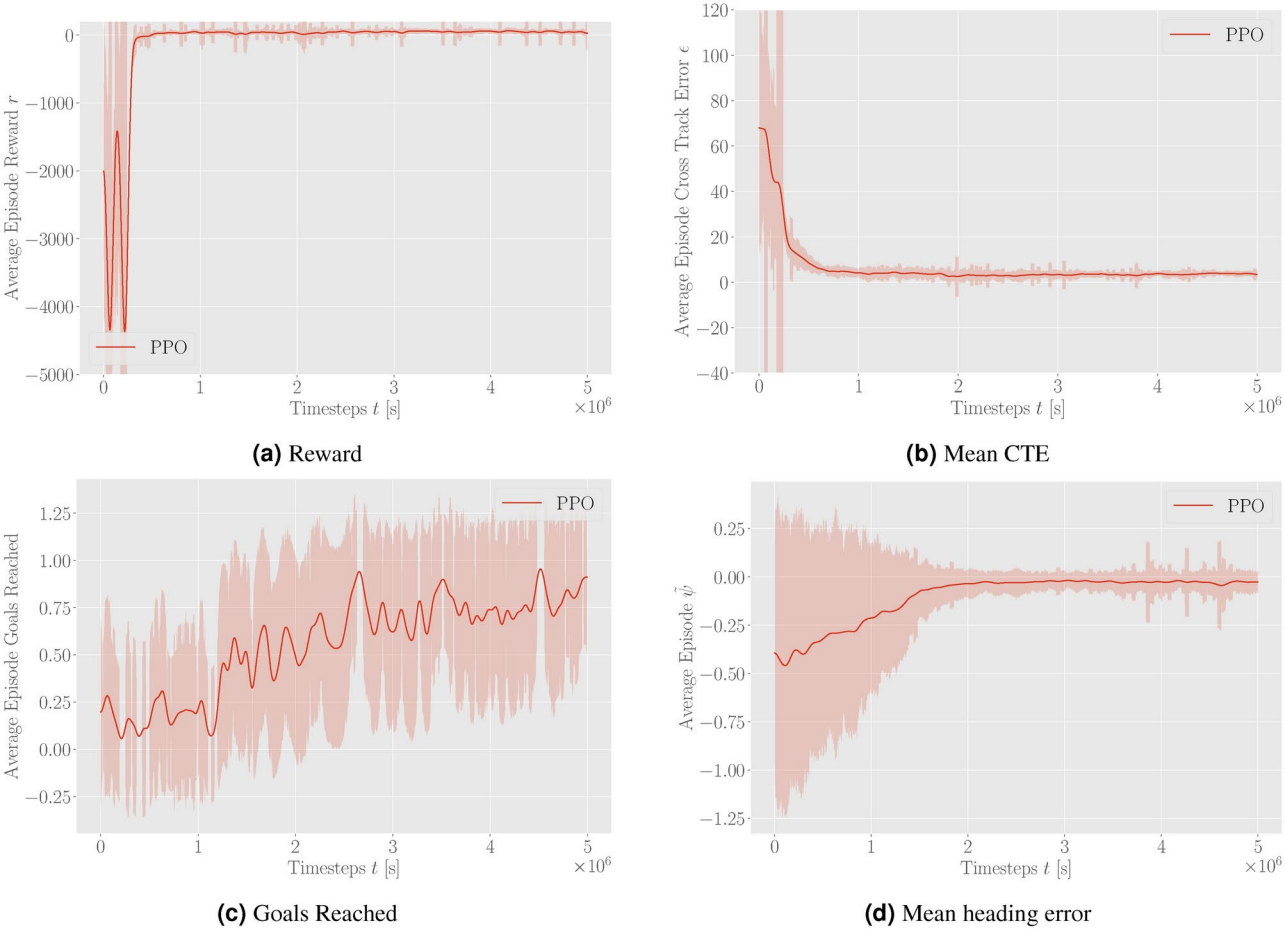
Average episode reward, CTE, goals reached, and heading error during training with the inclusion of environmental disturbances smoothed with a rolling average over 100 episodes.

For realistic representations, additional simulations were conducted, including environmental disturbances demonstrated in Fig. 5 to show the performance within more challenging training environments. In the presence of environmental disturbances, the advantages of DRL-tuned NMPC for path following become evident.

**Figure 6:**
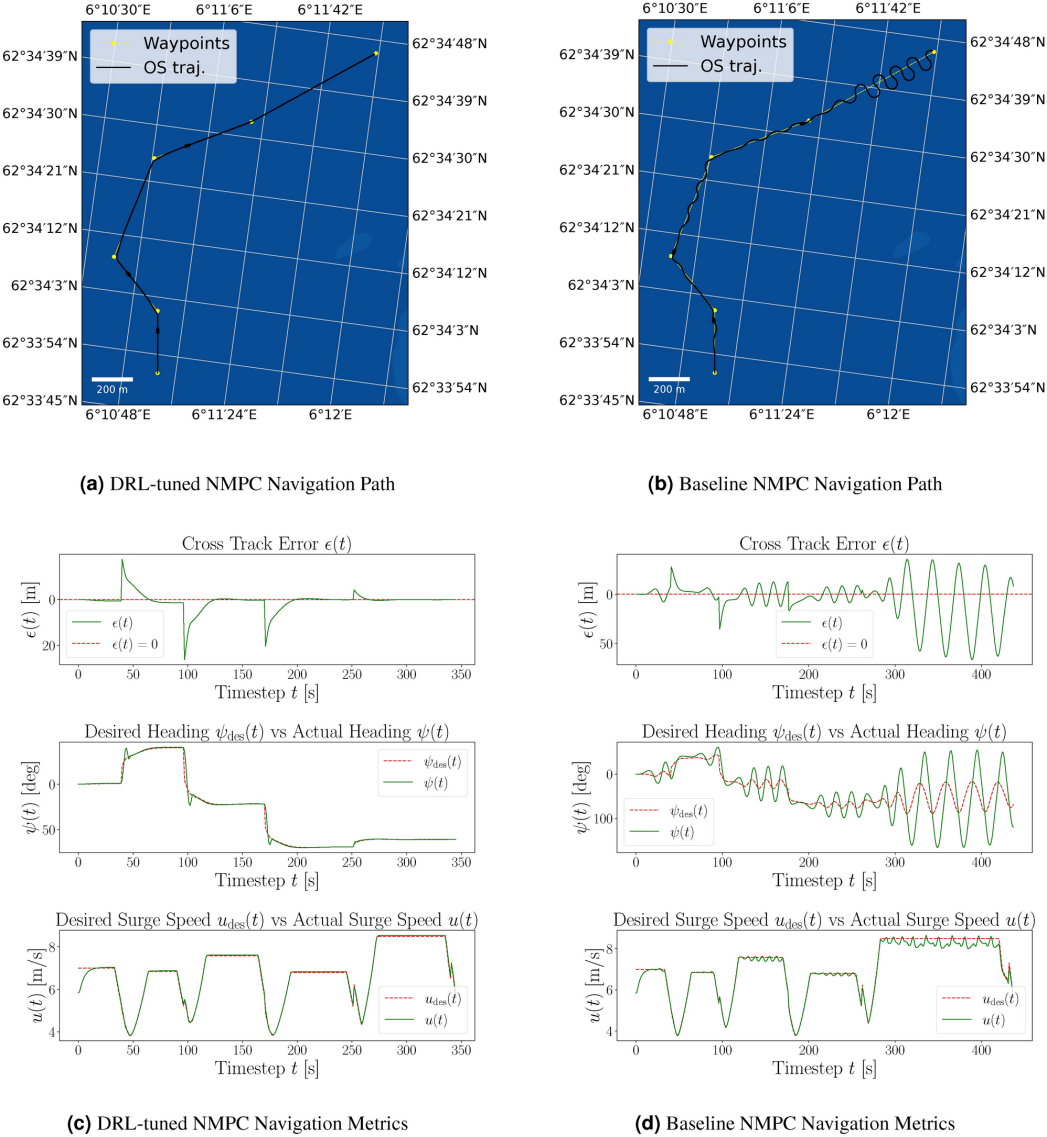
Comparison of path following with corresponding navigational metrics for the DRL-tuned and baseline NMPC in the same scenario, including environmental disturbances. The yellow dotted line indicates the desired path, and the black line shows the actual path of the ASV. It can be seen that the environmental forces from the northwest cause the baseline NMPC to exhibit oscillations around the desired path. At the same time, the DRL-tuned NMPC effectively compensates for the disturbances, maintaining accurate trajectory control through its feedback mechanism.

Key metrics show significant improvements. As demonstrated in Table 2, the average CTE is reduced to 2.90 meters from 14.47 meters, and the maximum CTE drops to 28.12 meters from 60.36 meters, indicating closer tracking to the desired path. The DRL-tuned NMPC also uses fewer time steps per episode and achieves goal-reaching performance in 94% of episodes, compared to 24% for the baseline, demonstrating enhanced navigational efficiency and robustness.

For heading control, the DRL-tuned NMPC maintains an average heading error near zero and reduces the maximum heading error to 0.54 degrees from 1.12 degrees, indicating precise directional control. With disturbances, the DRL-tuned NMPC shows clear benefits in surge speed control, reducing the average surge speed error to 0.04 meters per second from 0.21 meters per second, along with lower standard deviation and maximum surge speed errors, ensuring smoother and more accurate speed control.

Figure 6 illustrate the navigation paths and metrics in a scenario with environmental disturbances. The DRL-tuned NMPC (Fig. 6a) closely follows the desired path with minimal CTE, effectively compensating for disturbances. In contrast, the baseline NMPC (Fig. 6b) shows significant deviations and oscillations. Furthermore, Fig. 6c shows the DRL-tuned NMPC maintaining near-zero CTE between waypoints, highlighting the disturbance observer’s capability to compensate for environmental impact. The actual heading $\psi (t)$ closely follows the desired heading $\psi _{\text {des}}$ with minimal errors except around waypoints. Surge speed control is also precise, with minimal errors. The baseline NMPC, however, exhibits large oscillations and cross-track errors throughout (Fig. 6d), struggling to maintain the desired heading and overcorrecting frequently. This indicates inefficient heading control and slower error correction compared to the DRL-tuned NMPC.

**Table 2:** Comparison of PPO DRL agent and NMPC performance evaluated over 100 random episodes, with the inclusion of environmental disturbances.

Metric	DRL-tuned NMPC	Baseline NMPC
Episodes	100.00	100.00
Avg. cross-track error [m]	2.90	14.47
Avg. std cross-track error [m]	5.93	17.65
Avg. max cross-track error [m]	28.12	60.36
Avg. time steps [s]	353.97	401.05
Goals reached (%)	94.00%	24.00%
Avg. heading error [deg]	0.00	0.19
Avg. std heading error [deg]	0.11	0.66
Avg. max heading error [deg]	0.54	1.12
Avg. surge speed error [m/s]	0.04	0.21
Avg. std surge speed error [m/s]	0.24	0.26
Avg. max surge speed error [m/s]	1.73	1.78

#### COLAV performance

**Figure 7:**
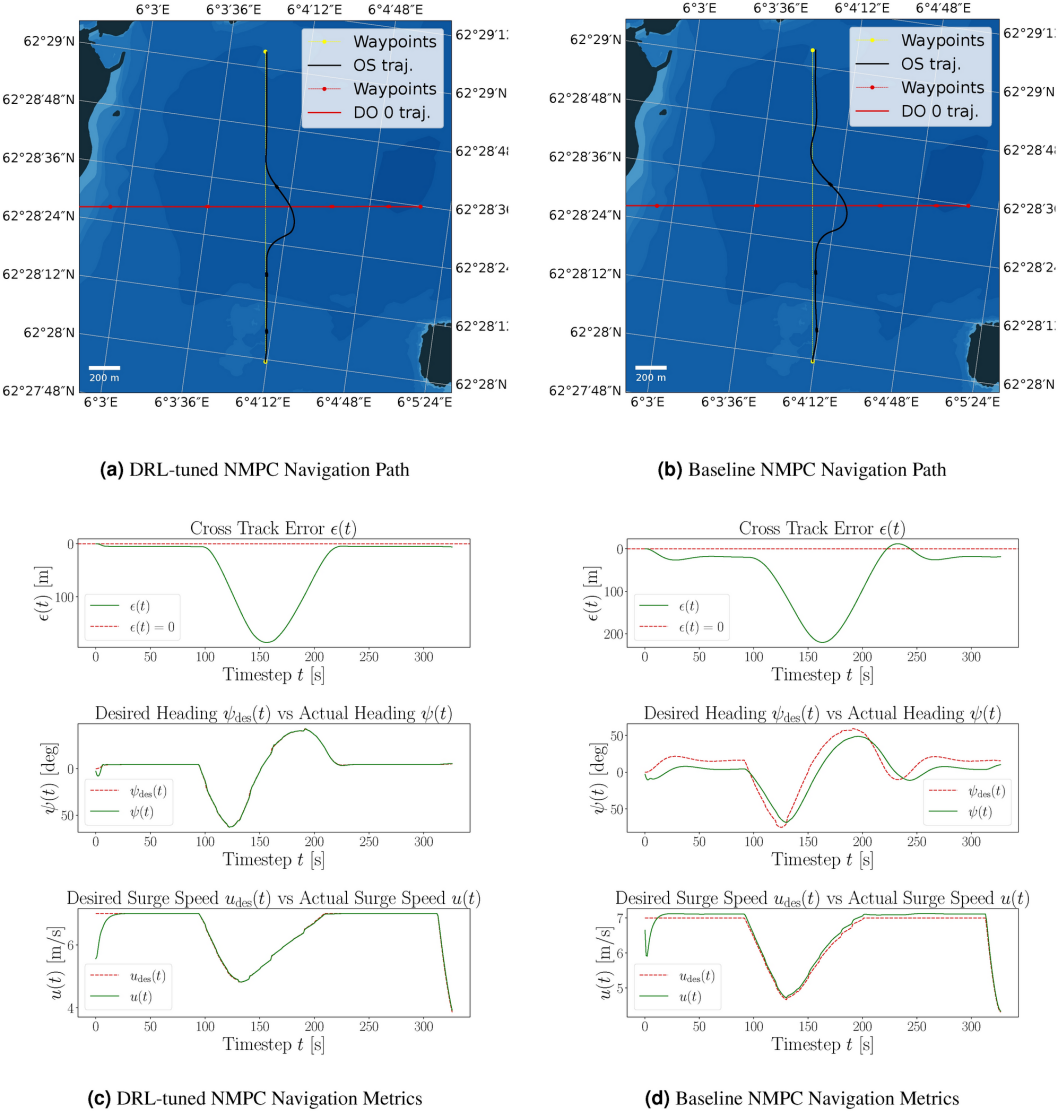
Comparison of navigation in a crossing give-way scenario, including disturbances for the DRL-tuned NMPC and the baseline NMPC, is depicted. The yellow dotted line indicates the desired path, the black line shows the actual path of the ASV avoiding a collision, and the red line depicts the trajectory of a crossing vessel.

The performance comparison of the DRL-tuned NMPC for a collision avoidance (crossing give way) scenario is presented in Fig. 7. The crossing give-way scenario assesses NMPC compliance and performance according to COLREG Rule 16^49^. Figure 7 displays navigation paths and metrics for the DRL-tuned NMPC and the baseline NMPC. Both controllers demonstrate COLREG compliance (Fig. 7a,b). The DRL-tuned NMPC maintains minimal CTE during straight paths, whereas the baseline NMPC shows slight overshoots.

Navigation metrics (Fig. 7c,d) reveal the DRL-tuned NMPC’s superior CTE and heading control. Furthermore, DRL-tuned NMPC exhibits marginally better surge speed control.

In Table 3, evaluation metrics are compared, demonstrating that the DRL-tuned NMPC achieves lower CTE during the crossing give-way maneuver, and completing the scenario in fewer time steps. It also shows reduced heading errors and a slightly improved surge speed control compared to the baseline NMPC.

**Table 3:** Comparison of evaluation metrics of DRL-tuned NMPC and baseline NMPC in the crossing give-way scenario, depicted in Fig. 7.

Metric	DRL-tuned NMPC	Baseline NMPC
Episodes	1.00	1.00
Avg. cross-track error [m]	7.91	18.54
Max cross-track error [m]	196.34	220.11
Time steps [s]	323.00	331.00
Avg. heading error [deg]	0.01	0.16
Max heading error [deg]	0.13	0.33
Avg. surge speed error [m/s]	0.06	0.09
Max surge speed error [m/s]	1.09	1.32

Overall, the DRL-tuned NMPC excels in path-following and heading control in the crossing give-way scenario, reflecting more efficient and accurate navigation. While both tuning approaches perform similarly, DRL proves beneficial for fine-tuning NMPC in specific scenarios, enhancing navigational efficiency and path following. This validation underscores the NMPC’s reliability and confirms compliance with COLREG, demonstrating its suitability for autonomous maritime navigation.

### DRL-tuned model parameters

Table 5 presents the mean, standard deviation, absolute error, and relative error for estimated matrices over 100 evaluation episodes compared to the actual ground truth values.

For the mass matrix ${\textbf{M}}$, estimates are generally accurate, with mean values close to actual values (e.g., ${3889.76}\text {kg}$ vs. ${3980.0}\text {kg}$ for the first diagonal element). However, some inaccuracies exist, such as the third diagonal element’s high standard deviation (${1140.86}\text {kgm}^{2}$) justified by the large ground truth value. Absolute errors range from ${90.24}\text {kgm}^{2}$ to ${137.01}\text {kgm}^{2}$, with relative errors mostly low, the highest being $3.44\%$.

The cubic damping matrix ${\textbf{D}}_{c}$ shows similar trends, with mean values close to the ground truth, especially the third diagonal element (3267.82 vs. 3224.0). However, variability is evident with a standard deviation of 155.37 for the third element. Absolute errors are small, the highest being 43.82, and relative errors are low, indicating overall high accuracy despite some fluctuation.

For the quadratic damping matrix ${\textbf{D}}_{q}$, the first element’s estimates are close to the ground truth (mean 147.82 vs. 135.0), with low standard deviations (2.73), and acceptable absolute errors, the highest being 12.82.

The linear damping matrix ${\textbf{D}}_{l}$ exhibits minimal deviation from actual values, with mean estimates like 54.15 vs. 50.0 for the first element. Standard deviations are low (1.45 for the first element), and absolute errors are small, the highest being 6.45, with minimal relative errors, confirming high estimation accuracy.

Table 4 shows the evaluation metrics for the model parameter estimation using the DRL agent trained over 10 million time steps, compared to the NMPC using the actual model parameters. The NMPC with DRL-tuned parameters achieves performance metrics comparable to those with actual model dynamics, demonstrating the DRL agent’s potential in effectively learning and adjusting model parameters.

**Table 4:** Evaluation metrics for NMPC using the estimated model parameters from the DRL agent in the state prediction of the NMPC, compared to the actual model parameters used for the state prediction.

Metric	DRL agent	Actual model parameters
Episodes	100.00	100.00
Avg. cross-track error	2.67	3.98
Avg. std cross-track error	3.38	3.64
Avg. max cross-track error	17.10	18.02
Avg. timesteps	203.71	203.92
Goals reached	97%	100.00%
Infeasible solutions	0.0	0.0
Avg. heading error	0.00	-0.01
Avg. mean heading error	0.00	-0.01
Avg. std heading error	0.09	0.08
Avg. max heading error	0.42	0.39
Avg. surge speed error	0.13	0.13
Avg. mean surge speed error	0.11	0.12
Avg. std surge speed error	0.39	0.39
Avg. max surge speed error	1.70	1.69

**Table 5:** Summary of mean, standard deviation (std), absolute error, and relative error of the estimated matrices and the actual ground truth values. The model is evaluated over 100 episodes drawn from separate test scenarios, with the same scenario generation configuration as the training. The training was executed by applying PPO with 10 million time steps.

Parameter	Mean	Std	Actual	Absolute error	Relative error (%)
${\textbf{M}}_{RB}$	$$\begin{bmatrix} 3889.76 & 0 & 0 \\ 0 & 3842.99 & 0 \\ 0 & 0 & 19783.01 \end{bmatrix}$$	$$\begin{bmatrix} 298.15 & 0 & 0 \\ 0 & 150.44 & 0 \\ 0 & 0 & 1140.86 \end{bmatrix}$$	$$\begin{bmatrix} 3980.0 & 0 & 0 \\ 0 & 3980.0 & 0 \\ 0 & 0 & 19703.0 \end{bmatrix}$$	$$\begin{bmatrix} 90.24 & 0 & 0 \\ 0 & 137.01 & 0 \\ 0 & 0 & 80.01 \end{bmatrix}$$	$$\begin{bmatrix} 2.27 & 0 & 0 \\ 0 & 3.44 & 0 \\ 0 & 0 & 0.41 \end{bmatrix}$$
${\textbf{D}}_{c}$	$$\begin{bmatrix} 0.03 & 0 & 0 \\ 0 & 0.06 & 0 \\ 0 & 0 & 3267.82 \end{bmatrix}$$	$$\begin{bmatrix} 0.05 & 0 & 0 \\ 0 & 0.03 & 0 \\ 0 & 0 & 155.37 \end{bmatrix}$$	$$\begin{bmatrix} 0.0 & 0 & 0 \\ 0 & 0.0 & 0 \\ 0 & 0 & 3224.0 \end{bmatrix}$$	$$\begin{bmatrix} 0.03 & 0 & 0 \\ 0 & 0.06 & 0 \\ 0 & 0 & 43.82 \end{bmatrix}$$	$$\begin{bmatrix} 0.03 & 0 & 0 \\ 0 & 0.06 & 0 \\ 0 & 0 & 1.36 \end{bmatrix}$$
${\textbf{D}}_{q}$	$$\begin{bmatrix} 147.82 & 0 & 0 \\ 0 & 2107.14 & 0 \\ 0 & 0 & -0.01 \end{bmatrix}$$	$$\begin{bmatrix} 2.73 & 0 & 0 \\ 0 & 109.51 & 0 \\ 0 & 0 & 0.02 \end{bmatrix}$$	$$\begin{bmatrix} 135.0 & 0 & 0 \\ 0 & 2000.0 & 0 \\ 0 & 0 & 0.0 \end{bmatrix}$$	$$\begin{bmatrix} 12.82 & 0 & 0 \\ 0 & 107.14 & 0 \\ 0 & 0 & 0.01 \end{bmatrix}$$	$$\begin{bmatrix} 9.50 & 0 & 0 \\ 0 & 5.36 & 0 \\ 0 & 0 & 0.01 \end{bmatrix}$$
${\textbf{D}}_{l}$	$$\begin{bmatrix} 54.15 & 0 & 0 \\ 0 & 199.52 & 0 \\ 0 & 0 & 1287.45 \end{bmatrix}$$	$$\begin{bmatrix} 1.45 & 0 & 0 \\ 0 & 6.98 & 0 \\ 0 & 0 & 90.35 \end{bmatrix}$$	$$\begin{bmatrix} 50.0 & 0 & 0 \\ 0 & 200.0 & 0 \\ 0 & 0 & 1281.0 \end{bmatrix}$$	$$\begin{bmatrix} 4.15 & 0 & 0 \\ 0 & 0.48 & 0 \\ 0 & 0 & 6.45 \end{bmatrix}$$	$$\begin{bmatrix} 8.29 & 0 & 0 \\ 0 & 0.24 & 0 \\ 0 & 0 & 0.50 \end{bmatrix}$$

The DRL agent yields a higher average reward with lower standard deviation than the actual model parameters, indicating effective optimization of the reward function. Furthermore, the average and mean CTE for the DRL agent are lower, showcasing better path-following accuracy, although the standard deviation of CTE is slightly higher, reflecting variability due to inaccurate model parameter estimates. The maximum CTE is lower for the DRL agent, indicating more precise path following.

The average time steps and duration per episode are nearly identical for both configurations, showing consistent efficiency in navigation tasks. Goal-reaching performance is perfect for the actual model parameters and slightly lower for the DRL agent, indicating reliable parameter settings by the DRL agent.

Both configurations show no infeasible solutions, confirming the viability of the DRL agent’s parameter settings in solving the NMPC’s OCP. The heading error metrics (average, mean, standard deviation, and maximum values) are very close to zero for both configurations, indicating precise heading control by the DRL agent. Surge speed error metrics are almost identical for both configurations, demonstrating the DRL agent’s ability to maintain the desired surge speed accurately.

The evaluation metrics suggest that the NMPC with the DRL agent setting model parameters achieves similar performance to using actual model parameters. This highlights the DRL agent’s success in setting model parameters for efficient and safe NMPC operation, despite inaccuracies in parameter estimates. This approach shows promise for real-time model parameter identification, although further refinement is needed for accurate physical dynamics capture. The robustness of the NMPC, even with suboptimal model parameters, underscores its potential application in real-world scenarios.

Traditional NMPC methods often provide formal stability guarantees, ensuring predictable and safe behavior under various operating conditions. In contrast, DRL lacks inherent theoretical guarantees for stability. In addition, the effectiveness of DRL depends heavily on the design of the reward function, which guides the learning process. An improperly designed reward function can lead to unintended or suboptimal behavior, such as prioritizing energy efficiency at the expense of safety during trajectory tracking. For ASVs, where safety and operational efficiency must be carefully balanced, crafting an appropriate reward function is crucial. Finally, it is important to note that the decision-making processes of DRL models, especially those relying on deep neural networks, are often intransparent and difficult to interpret. Therefore, the synergy of DRL and NMPC may reduce these weaknesses, enabling a reliable and adaptable control approach for dynamic maritime environments.

## Conclusions

The integration of deep reinforcement learning (DRL) with nonlinear model predictive control (NMPC), as explored in this study, holds significant relevance in the context of digital twins. By utilizing DRL for the optimization of NMPC parameters and real-time model parameter identification, this work enhances the accuracy, reliability, and adaptability of digital twins. As shown in Table 1, the DRL-optimized NMPC reduces the average cross-track error by more than fourfold and achieves a 100% waypoint success rate, compared to just 27% with the manually tuned NMPC. Furthermore, as demonstrated in Table 5, the actual system model is approximated with a maximum relative parameter error below 10% and an average relative parameter error of approximately 2.6%. In the case of autonomous surface vessels (ASVs), the proposed approach allows for continuous updating of the digital twin, ensuring it remains an accurate representation of the physical vessel under dynamic and complex maritime conditions. This continuous updating process mitigates the inevitable drift that occurs when models are static and unresponsive to real-time changes. By learning and refining control strategies within the virtual environment of the digital twin, the study demonstrates how it is possible to optimize control performance without exposing the physical asset to risk. Consequently, this work not only contributes to advancing the functionality and utility of digital twins but also provides a robust framework for their application in enhancing the safety, efficiency, and performance of autonomous systems in real-world scenarios. The framework can potentially be extended with additional constraint satisfaction mechanisms, such as predictive safety filters, to enforce safety within NMPC constraints. Moreover, predictive awareness of weather and dynamic objects can enhance guidance and path planning, further mitigating environmental risks in maritime operations. To finally assess the effectiveness of the proposed framework under practical conditions, real-world validation tests would be essential.

## Data Availability

The datasets used and/or analysed during the current study available from the corresponding author on reasonable request.
